# Engineered *Bifidobacterium* Strains Colonization at Tumor Sites: A Novel Approach to the Delivery of Cancer Treatments

**DOI:** 10.3390/cancers17152487

**Published:** 2025-07-28

**Authors:** Rhea Amonkar, Ashley Ann Uy, Pablo Ramirez, Harina Patel, Jae Jin Jeong, Nicole Oyinade Shoyele, Vidhi Vaghela, Ashakumary Lakshmikuttyamma

**Affiliations:** Department of Pharmaceutical Sciences, Jefferson College of Pharmacy, Thomas Jefferson University, Philadelphia, PA 19107, USA

**Keywords:** *Bifidobacterium*, drug delivery, cancer treatments, gene therapy, immunotherapy, HIFU synergist, cancer hypoxia

## Abstract

Bacteria-mediated cancer therapy represents a novel and promising strategy for targeted drug delivery to solid tumors. Multiple studies have demonstrated that various *Bifidobacterium* species can selectively colonize the hypoxic microenvironments characteristic of solid tumors. Leveraging this property, *Bifidobacterium* has been explored as a delivery vector for a range of anti-cancer approaches such as immunotherapy, nanoformulated chemotherapeutics, and gene therapy. Notably, anti-angiogenic genes such as endostatin and tumstatin have been successfully delivered to colorectal tumors using *Bifidobacterium infantis* and *Bifidobacterium longum*, respectively. Additionally, *Bifidobacterium bifidum* has been employed to transport doxorubicin and paclitaxel nanoparticles to breast and lung tumor sites. Furthermore, both *Bifidobacterium longum* and *Bifidobacterium bifidum* have been utilized to deliver nanoparticles that act as synergistic agents for high-intensity focused ultrasound (HIFU) therapy, significantly enhancing tumor ablation, particularly in triple-negative breast cancer (TNBC) models. While these pre-clinical findings are highly encouraging, further clinical research is essential. Specifically, studies are needed to investigate the colonization dynamics of different *Bifidobacterium* species across various tumor types and to evaluate their potential in delivering diverse cancer therapies in human patients.

## Simple Summary

Gut microbes have demonstrated the ability to suppress cancer growth and prevent its spread to other organs. Moreover, certain gut microbes can enhance the effectiveness of immunotherapy. This review focuses on the use of a specific gut microbe, *Bifidobacterium*, as a vehicle for targeted cancer therapy. Researchers have successfully employed *Bifidobacterium* to deliver various treatments such as antibody therapies, chemotherapeutic agents, gene therapies, and enhancers for high-intensity focused ultrasound directly to tumor sites. This targeted approach not only boosts the anticancer efficacy of these treatments but also minimizes their associated side effects. Although these promising results have been observed in animal models, further clinical studies are essential to validate the potential of this innovative therapeutic strategy in humans.

## 1. Introduction

Cancer remains one of the leading causes of death worldwide, contributing significantly to global mortality rates. Conventional treatment modalities such as surgery, chemotherapy, and radiation therapy have long been employed to manage tumor growth and metastasis. While these approaches are effective for many patients, their non-specific nature can result in the occurrence of unwanted side effects. In response, the pursuit of more targeted therapies has led to the development of monoclonal antibody treatments and nanoparticle-based drug delivery systems [1,2,3,4,5,6,7,8]. These advanced therapeutic strategies have markedly improved cancer-related outcomes. By enabling the targeted delivery of nano-formulated anticancer agents and monoclonal antibody or protein therapies directly to tumor sites, these innovations enhance treatment efficacy while minimizing systemic side effects. Recent studies have increasingly focused on the use of bacterial vectors for delivering therapeutic agents into the tumor micro-environment. These vectors have demonstrated the ability to transport single or multiple therapeutic agents directly to solid tumor sites [9]. Compared to viral vectors, bacterial vectors offer several advantages: they can actively target tumors and penetrate deep into tissues, thereby enhancing the delivery of therapeutics to cancer cells. Moreover, bacterial vectors do not integrate their genetic material into the host genome, which reduces the risk of mutagenesis and potential oncogenesis [10].

Various bacterial species, including *Salmonella*, *Escherichia coli* (*E. coli*), *Listeria*, and photosynthetic bacteria, have been explored for cancer drug delivery applications [11]. Notably, anaerobic bacteria have gained attention due to their ability to colonize hypoxic regions within solid tumors. Among them, *Bifidobacterium*, an anaerobic bacterium, is being actively investigated for its potential to deliver a range of anticancer agents directly to tumor sites.

## 2. *Bifidobacterium* and Hypoxic Environment

Hypoxia is a hallmark characteristic of solid tumors. The partial pressure of oxygen (pO_2_) in tumor tissues typically ranges from 10 to 30 mmHg, in contrast to 24–66 mmHg in normal tissues. This oxygen deficiency arises from the rapid and uncontrolled proliferation of tumor cells, which leads to an elevated demand for oxygen that outpaces supply [12]. Tumor hypoxia plays a critical role in modulating the efficacy of various anticancer therapies, including radiotherapy, chemotherapy, and immunotherapy [13,14]. Although the hypoxic tumor microenvironment presents significant challenges for conventional cancer treatments, it also offers unique opportunities for targeted drug delivery. Notably, the low-oxygen conditions create a favorable niche for the colonization and proliferation of anaerobic bacteria, which can be harnessed for site-specific therapeutic delivery.

Bacteria such as *Bifidobacterium*, *Salmonella, Escherichia coli* (*E. coli*), and *Clostridium perfringens* have demonstrated the ability to inhibit cancer cell growth by selectively colonizing tumor tissues. In addition to their natural tumor-targeting capabilities, several of these anaerobic species have been engineered or utilized to deliver a variety of anticancer agents directly to malignant cells [15]. Among them, both wild-type and genetically modified strains of *Bifidobacterium* have been widely studied for their potential in targeted drug delivery across different types of cancer. Unlike conventional therapies that target specific receptors or proteins, *Bifidobacterium*-mediated delivery systems are designed to target the entire tumor mass, leveraging the unique microenvironment of solid tumors for more comprehensive therapeutic coverage.

One of the other factors that assist in the colonization of bifidobacteria in cancer cells is its acid resistance. Due to this property, *Bifidobacterium* can grow in the acidic PH [16]. Most cancer cells often exhibit an acidic extracellular pH compared to normal cells. This acidic environment is primarily due to increased glycolysis, which generates lactate and H+, creating an acidic environment within tumors [17].

## 3. *Bifidobacterium* Colonization in Tumors

A substantial body of research has demonstrated the ability of *Bifidobacterium* species to selectively colonize various tumor types. In an early study, Kimura et al. [18] reported that intravenous administration of *Bifidobacterium bifidum* resulted in its localization and proliferation within mouse tumors, with no detectable presence in normal tissues. Furthermore, the growth of *Bifidobacterium bifidum* was enhanced by the administration of lactulose (4-O-β-D-galactopyranosyl-D-fructofuranose) [18]. Similarly, Yasawa et al. observed that systemically administered wild-type *Bifidobacterium* longum selectively localized and proliferated in multiple solid tumor models, while remaining undetectable in healthy organs such as the kidney, spleen, and liver [19]. Another study demonstrated that a genetically modified strain, *Bifidobacterium breve* UCC2003, successfully colonized in B16-F10 murine melanoma tumors [20]. The study also found that levels of *Bifidobacterium* breve were significantly elevated in tumors, particularly in those lacking T cells, following both oral and intravenous administration.

A pre-clinical study using a murine model of Lewis lung carcinoma demonstrated that *Bifidobacterium longum* strains 105A and 108A, when administered via tail vein injection, selectively colonized in tumor tissues [19]. The authors observed that genetically engineered *Bifidobacterium longum* localized exclusively within tumor sites 96 to 168 h post-injection, with no detectable presence in healthy organs such as the liver, spleen, or kidneys. Moreover, the modified bacteria were capable of proliferating within the tumor microenvironment. This selective colonization is likely attributed to the hypoxic condition characteristic of solid tumors, which provides a favorable niche for the growth of anaerobic bacteria like *Bifidobacterium* [19].

An in vitro study demonstrated the tumor-targeting potential of *Bifidobacterium* using a three-dimensional (3D) multicellular tumor spheroid (MCTS) model. This model was employed to evaluate the feasibility of prodrug-converting enzyme therapy via bacterial vectors. When *Bifidobacterium bifidum* S17 was incubated with HT-29-derived MCTS, the bacteria localized, survived, and replicated within the hypoxic core of the spheroids. To assess the persistence of the bacteria, colony-forming units were measured up to 72 h post-infection. Results indicated that bacterial levels remained stable within the MCTS, whereas in standard culture media without MCTS, bacterial counts declined significantly within the first 24 h. These findings suggest that HT-29 MCTS provides a suitable in vitro platform for studying tumor-targeting strategies involving anaerobic bacteria [21]. Additionally, Yazawa et al. reported that genes with anticancer activity can be cloned into the pBLES100 vector, expressed in *Bifidobacterium longum*, and selectively delivered to tumor tissues [19].

*Bifidobacterium* has also been explored as a delivery vehicle for different treatment options such as gene therapy, chemotherapeutic agents, Immunotherapy, and nanoparticle-formulated drugs, highlighting its versatility in cancer therapy.

## 4. *Bifidobacterium*-Mediated Gene Therapy

The efficacy of gene therapy largely depends on the specificity and efficiency of the gene delivery system. The study by Yazawa et al. underscores the challenges of targeting solid tumors and highlights the potential of *Bifidobacterium longum* as a promising solution due to its selective localization and proliferation within tumor tissues. Using a dimethylbenz[a]anthracene-induced rat mammary tumor model, the researchers demonstrated the feasibility of employing genetically engineered *Bifidobacterium longum* as a tumor-specific gene delivery vector for breast cancer [22]. 5-Fluorouracil (5-FU) is a widely used chemotherapeutic agent for breast cancer; however, achieving therapeutic concentrations at the tumor site typically requires high systemic doses, which can lead to significant toxicity. To address this, Fujimori and colleagues developed the Bifidobacterial selective targeting Cytosine Deaminase (BEST-CD) system. In this approach, the cytosine deaminase gene was inserted into *Bifidobacterium longum*, enabling the conversion of the non-toxic prodrug 5-fluorocytosine (5-FC) into active 5-FU directly within the tumor. Their findings revealed that high concentrations of 5-FU were detected exclusively in mammary tumor tissues, with no presence in normal organs, supporting the use of *Bifidobacterium longum* for enzyme/prodrug therapy in hypoxic tumors [23,24]. Further research by the same group compared the efficacy of *Bifidobacterium breve*, a smaller species, with *Bifidobacterium longum* in delivering enzyme/prodrug therapy in a murine lung cancer model. Both strains were transformed with a plasmid encoding cytosine deaminase via electroporation. Interestingly, *Bifidobacterium breve* exhibited higher enzymatic activity for converting 5-FC to 5-FU compared to *Bifidobacterium longum*, suggesting its potential as a more efficient vector for targeted cancer therapy [25].

Endostatin, an anti-angiogenic factor, inhibits the basic fibroblast growth factor (bFGF)-mediated proliferation of vascular endothelial cells in solid tumors. In this study, researchers utilized a strain of *Bifidobacterium adolescentis* as a delivery vector to transport the endostatin gene into solid tumors. This bacterium selectively colonized in the hypoxic microenvironment of tumor tissues, leading to a reduction in angiogenesis and tumor growth in liver cancer models [26]. Tumstatin, another angiogenesis inhibitor, was shown to inhibit the PI3K signaling pathway, increase caspase-3 activity, promote apoptosis in vascular endothelial cells, and suppress tumor growth [27]. In murine models of colon carcinoma, an engineered *Bifidobacterium longum* expressing tumstatin was administered via three different routes: intratumoral injection (INT-BL-Tum), intravenous injection through the vena caudalis (INV-BL-Tum), and oral delivery (OR-BL-Tum). At the end of the experimental period, tumor growth inhibition was observed at 38.56% for OR-BL-Tum, 75.21% for INT-BL-Tum, and 64.63% for INV-BL-Tum [28].

Claudin-4, a transmembrane protein involved in tight junctions, is highly overexpressed in triple-negative breast cancer (TNBC). The expression of claudin-4 can be selectively inhibited by the C-terminal fragment of *Clostridium perfringens* enterotoxin (C-CPE). In this study, a genetically engineered strain of *Bifidobacterium longum*, designated *B. longum*-C-CPE-PE23, was developed to secrete the claudin-4-targeting fusion protein C-CPE-PE23. Administration of *B. longum*-C-CPE-PE23 in a TNBC mouse model resulted in significant tumor growth inhibition compared to control groups. Notably, the treatment exhibited minimal systemic toxicity, highlighting its potential as a targeted and safe therapeutic strategy for TNBC [29].

Interleukin-24 (IL-24) is known to regulate the growth of solid tumors by inducing apoptosis and/or autophagy. It has been widely recognized for its anti-cancer properties, primarily through the induction of apoptosis. IL-24-expressing *Bifidobacterium breve* (designated pLW5-breve) was developed to evaluate its therapeutic potential in a mouse model of head and neck squamous cell carcinoma. Recombinant *Bifidobacterium breve*-IL24 treatment exhibited significantly reduced tumor growth compared to the control group (*Bifidobacterium breve*-GFP). Further tumor tissue analysis revealed that *B. breve*-IL24 treatment led to decreased expression of the anti-apoptotic protein BCL-2 and increased expression of the pro-apoptotic protein Bim, supporting its role in promoting tumor cell apoptosis [30].

**Figure 1:**
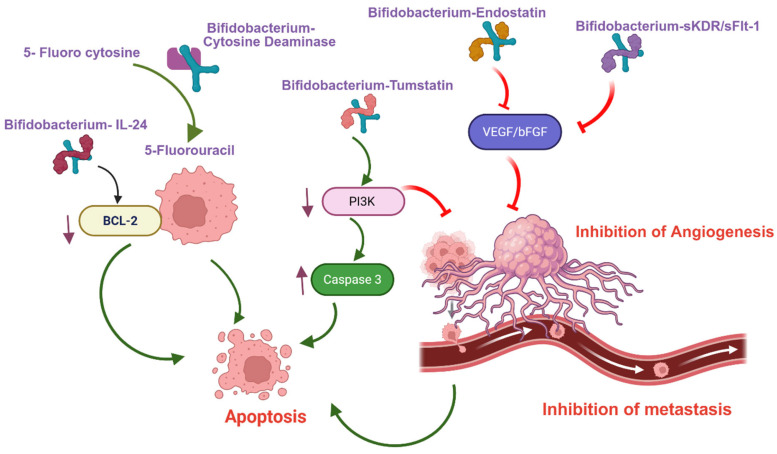
Schematic representation of *Bifidobacterium*-facilitated gene therapy delivery and its signaling mechanisms in suppressing tumor metastasis and promoting apoptosis.

VEGF-mediated angiogenesis can be inhibited by soluble forms of tyrosine kinase receptors that bind VEGF, such as the fms-like tyrosine kinase receptor (sFlt-1) and the kinase insert domain receptor (sKDR) [31]. *Bifidobacterium infantis*-mediated prokaryotic expression system was developed to deliver the *sKDR* gene. In vitro studies using human umbilical vein endothelial cells (HUVECs) demonstrated that this system effectively downregulated VEGF expression. Furthermore, in a Lewis lung carcinoma mouse model, B. infantis-sKDR treatment significantly reduced angiogenesis and exhibited notable anti-tumor effects and increased the survival [32]. In a separate study, a *Bifidobacterium infantis*-mediated sFlt-1 gene delivery system was evaluated in the same lung cancer model. This approach also showed promising results, including significant inhibition of VEGF-induced HUVEC proliferation, reduced tumor growth, and prolonged survival in treated mice. These findings underscore the potential of *Bifidobacterium infantis*-based gene delivery systems as targeted anti-angiogenic therapies for cancer [33]. Table 1 summarizes *Bifidobacterium*-mediated gene therapy approaches. Figure 1 displays the schematic representation of the mechanism of tumor growth inhibition and metastasis by *Bifidobacterium*-mediated gene therapy delivery approaches.

## 5. *Bifidobacterium*-Mediated Immunotherapy

Genetically engineered *Bifidobacterium* longum to express cytosine deaminase that converts 5-fluorocytosine (5-FC) to 5-fluorouracil has been known as APS001F. Shioya et al. experimented with APS001F together with 5-FC and anti-PD-1 monoclonal antibody in the CT26 colorectal cancer mouse model. The combination therapy of APS001F/5-FC and anti-PD-1 monoclonal antibody (mAb) demonstrated significantly greater anti-cancer efficacy compared to either treatment alone, resulting in prolonged survival. Notably, this combination therapy led to a marked reduction in CD4^+^ T cells, an effect not observed in the monotherapy groups. Meanwhile, the proportion of CD8^+^ T cells remained stable across all treatment groups. These findings suggest that the combined APS001F/5-FC and anti-PD-1 mAb therapy not only enhances anti-tumor activity but also exerts immunomodulatory effects [34].

Trastuzumab, a monoclonal antibody targeting the human epidermal growth factor receptor 2 (HER2), is a standard therapeutic agent for HER2-positive breast cancer. To enhance its delivery to hypoxic tumor regions, researchers developed an innovative delivery system utilizing *Bifidobacterium longum*. Initially, a genetically engineered single-chain variable fragment (scFv) of trastuzumab was constructed. This was followed by the development of *Bifidobacterium longum* strains (designated H1 and H2) capable of expressing and secreting the trastuzumab scFv directly within the tumor microenvironment. The engineered *Bifidobacterium* strains demonstrated significant inhibition of HER2-positive breast cancer cell growth both in vitro and in vivo, highlighting their potential as a targeted therapeutic platform for hypoxic tumors [35].

Chlorin e6 (Ce6) is a photosensitizer known for its ability to induce cancer cell apoptosis by inhibiting immune checkpoints such as programmed cell death protein 1 (PD-1) and its ligand PD-L1. Under laser light or ultrasound (US) irradiation, Ce6 generates reactive oxygen species (ROS), leading to cancer cell death [36]. Additionally, death receptor 5 (DR5) is commonly overexpressed in many cancer types, making it a promising therapeutic target. To enhance tumor targeting and therapeutic efficacy, a multifunctional nanoparticle system was developed by conjugating Ce6 to *Bifidobacterium bifidum*, followed by attachment of an anti-DR5 antibody, resulting in the construct Ce6–*B. bifidum*–anti-DR5 Ab. In a laryngeal cancer mouse model, combined treatment with Ce6–*B. bifidum*–anti-DR5 Ab and sequential US and laser irradiation (Ce6–*B. bifidum*–anti-DR5 Ab + US→Laser) led to complete tumor regression within six days. In contrast, the group treated with Ce6–*B. bifidum* + US→Laser showed only partial tumor inhibition. These findings highlight the synergistic role of both anti-DR5 antibody targeting and *Bifidobacterium bifidum*-mediated tumor localization in enhancing the anti-cancer efficacy of Ce6-based photodynamic and sonodynamic therapies [37].

## 6. *Bifidobacterium*-Mediated Nanoparticle Formulated Chemotherapy Delivery

While oral chemotherapy drugs have been shown to effectively reduce tumor growth and metastasis, their lack of tumor-specific targeting often results in toxicity to healthy tissues. Doxorubicin (also known as Adriamycin) is a widely used chemotherapeutic agent for treating various solid tumors, including breast cancer. However, its clinical utility is significantly limited by its associated cardiotoxicity. In recent years, nanoparticle-based formulations of chemotherapeutic agents have gained considerable attention due to their ability to provide sustained drug release, enhanced stability, and improved therapeutic index through more targeted delivery.

A recent study developed a biohybrid drug delivery system using *Bifidobacterium infantis* to transport doxorubicin-loaded nanoparticles to hypoxic breast tumors. This system, termed Bif@BDC-NPs, consists of *Bifidobacterium infantis* combined with albumin-encapsulated doxorubicin nanoparticles coated with chitosan. In an in vivo breast cancer model, Bif@BDC-NPs effectively targeted the tumor site and achieved a significant 94% inhibition of tumor growth, while also reducing the adverse effects typically associated with doxorubicin treatment [38]. Similarly, another study employed *Bifidobacterium infantis* to deliver doxorubicin-loaded bovine serum albumin nanoparticles (DOX-NPs). Treatment with this biohybrid (Bif@DOX-NPs) resulted in a fourfold increase in doxorubicin concentration at the tumor site compared to treatment with doxorubicin alone. Additionally, the biohybrid extended the median survival of the breast cancer model to 69 days and mitigated the drug’s systemic toxicity [15].

**Figure 2:**
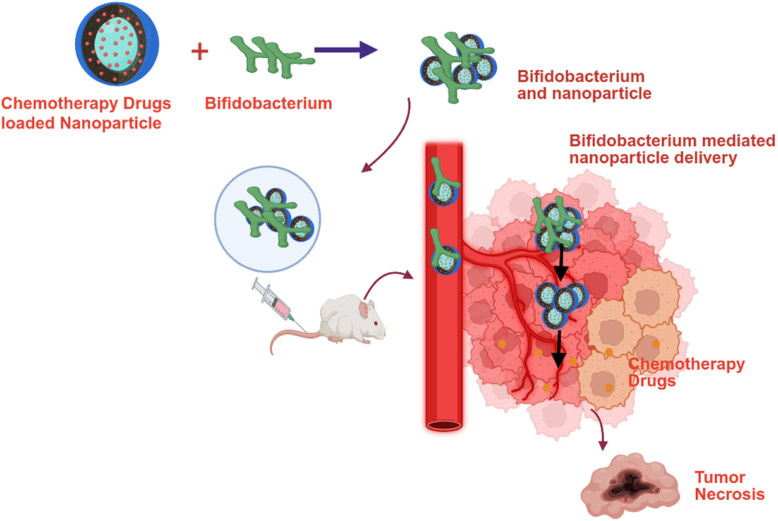
Depiction of *Bifidobacterium*-mediated delivery of nano-formulated chemotherapeutic agents within the tumor microenvironment, leading to the induction of tumor necrosis.

Paclitaxel is a widely used chemotherapeutic agent for the treatment of various solid tumors. In a study by Shi et al. [39], polydopamine (PDA)-coated paclitaxel nanoparticles were developed and incorporated into a *Bifidobacterium infantis*-based biohybrid delivery system, termed Bif@PDA-PTX-NPs. This biohybrid was evaluated in a lung cancer model, where it demonstrated sustained release of paclitaxel nanoparticles specifically at the tumor site. The anti-tumor efficacy of Bif@PDA-PTX-NPs was significantly greater than that of non-formulated paclitaxel. The authors concluded that this biohybrid system represents a promising and novel nanoparticle-based therapeutic strategy for lung cancer and potentially other solid tumors [39]. The anti-cancer activity of irinotecan (CPT-11) is primarily mediated through its active metabolite, 7-ethyl-10-hydroxycamptothecin (SN-38). However, SN-38 is associated with severe systemic toxicity due to its poor tumor-targeting capability. Although various nano-formulations of SN-38 have been developed, achieving effective drug localization within the hypoxic tumor microenvironment remains a significant challenge. To address this, a study developed a water-soluble prodrug, poly-L-glutamic acid-conjugated SN-38 (L-PGA-SN38), which was then complexed with *Bifidobacterium bifidum*. The resulting formulation, referred to as CS-L-PGA-SN38 NPs/B. bifi, demonstrated targeted delivery of SN-38 to tumor sites. In a colorectal cancer mouse model (HT-29), this biohybrid system achieved an impressive 80% tumor growth inhibition, highlighting its potential as an effective and targeted therapeutic strategy [40]. Another study developed a biological hybrid drug delivery system, BI-ES-FeAlg/DOX, using nano-sized iron alginate (FeAlg) gel with doxorubicin, endostatin, *and Bifidobacterium infantis*. Both in vitro and in vivo experiments confirmed the effective delivery of endostatin and doxorubicin to cancer cells, resulting in the suppression of tumor metastasis through downregulation of bFGF and VEGF expression. This study found that the *Bifidobacterium infantis*-based hybrid system offers a promising platform for the combined delivery of chemotherapeutic agents and anti-angiogenic genes to colorectal tumors [41]. Table 2 summarizes the *bifidobacterium*-mediated delivery of nano-formulated chemotherapy agents. Figure 2 represents the schematic representation of the nanoparticle-formulated chemotherapy delivery to cancer cells via *bifidobacterium*.

## 7. *Bifidobacterium*-Mediated HIFU Synergistic Nanoparticles Delivery

High-intensity focused ultrasound (HIFU) is an emerging non-invasive technique for the ablation of localized tumors. It has been successfully applied in the treatment of various solid tumors, including those of the breast, liver, pancreas, and prostate. Compared to conventional surgery and radiation therapy, HIFU is associated with fewer complications [42]. However, ongoing research aims to enhance its therapeutic efficacy using HIFU synergistic agents, which can significantly improve treatment outcomes. Crucially, the effective localization of these synergistic agents at the tumor site is essential for maximizing HIFU’s anti-cancer potential. In a recent study, a bio-targeted nanoparticle system was developed for HIFU-enhanced therapy in triple-negative breast cancer (TNBC). The researchers engineered a HIFU synergistic nanoparticle, PFH@CL/Fe_3_O_4_, composed of perfluorohexane (PFH), superparamagnetic iron oxide nanoparticles (Fe_3_O_4_), and cationic lipids (CL). *Bifidobacterium bifidum* was employed as a delivery vehicle to transport these nanoparticles to the TNBC tumor site via electrostatic adsorption. In vitro and in vivo experiments demonstrated that *Bifidobacterium bifidum* effectively colonized the hypoxic tumor microenvironment. The combination treatment group receiving B. bifidum + PFH@CL/Fe_3_O_4_ NPs + HIFU exhibited significantly greater tumor necrosis volume compared to the group treated with HIFU alone. These findings suggest that *Bifidobacterium bifidum*-mediated delivery of HIFU synergistic agents represents a promising and effective therapeutic strategy for TNBC [43].

Another group of researchers also explored the tumor-targeting potential of *Bifidobacterium bifidum* to enhance the efficacy of HIFU therapy in TNBC. They developed an aptamer-directed nanoparticle system, AP-PFH/PLGA, consisting of perfluorohexane (PFH)-loaded poly (lactic-co-glycolic acid) (PLGA) nanoparticles functionalized with the aptamer CCFM641-5. *Bifidobacterium bifidum* was employed to deliver these nanoparticles to the tumor microenvironment. The study demonstrated that *Bifidobacterium bifidum* successfully colonized the tumor site, facilitating the targeted delivery of AP-PFH/PLGA nanoparticles. Following HIFU ablation, the group treated *B. bifidum* + AP-PFH/PLGA exhibited the highest level of coagulative necrosis, indicating enhanced therapeutic efficacy. The findings suggest that this aptamer-guided, *Bifidobacterium bifidum*-mediated approach significantly improves the effectiveness of HIFU in TNBC treatment [44]. In a separate study aimed at enhancing HIFU efficacy, researchers utilized *Bifidobacterium longum* to deliver PEGylated cationic lipid nanoparticles (CL-NPs) in a TNBC model. The combination of *Bifidobacterium longum* and CL-NPs significantly improved the therapeutic efficiency of HIFU. The group receiving HIFU + *B. longum* + CL-NPs showed a greater ablation effect and a larger volume of coagulative necrosis compared to the group treated with HIFU + CL-NPs alone. These results highlight the strong synergistic effect of *Bifidobacterium*-mediated nanoparticle delivery in enhancing HIFU-based anti-cancer therapy for TNBC [45].

Another research group leveraged the tumor-homing capabilities of *Bifidobacterium bifidum* to deliver polyethylenimine (PEI)-modified poly (lactic-co-glycolic acid) nanoparticles loaded with sodium bicarbonate (PEI-PLGA-NaHCO_3_ NPs) in a TNBC mouse model. The study demonstrated that *Bifidobacterium bifidum*-conjugated nanoparticles successfully colonized the tumors in 4T1 breast cancer-bearing BALB/c mice. Notably, the presence of PEI significantly enhanced the electrostatic interaction between the nanoparticles and *Bifidobacterium bifidum*, resulting in a high adsorption rate of 96.32% for PEI-PLGA-NaHCO_3_ NPs, compared to just 5.11% for unmodified PLGA-NaHCO_3_ NPs. Further analysis revealed that the combination treatment group receiving HIFU + *B. bifidum* + PEI-PLGA-NaHCO_3_ NPs exhibited greater tumor nuclear fragmentation and dissolution than the group treated with HIFU alone. Additionally, the required ultrasound energy for effective HIFU ablation was reduced due to the cavitation nuclei generated by the nanoparticles, which enhanced the HIFU-mediated tumor destruction. These findings highlight the synergistic potential of PEI-PLGA-NaHCO_3_ NPs in improving the efficacy of HIFU therapy for TNBC through *Bifidobacterium bifidum*-mediated targeted delivery [46].

A recent study focused on developing a multifunctional biological agent capable of tumor targeting, enhancing high-intensity focused ultrasound (HIFU) therapy, and enabling multimodal imaging for precise treatment guidance. Researchers constructed a synergistic therapeutic platform using *Bifidobacterium longum* as a biological carrier, combined with cationic lipid nanoparticles co-loaded with indocyanine green (ICG) and perfluorohexane (PFH), referred to as CL-ICG-PFH-NPs. These nanoparticles were electrostatically adsorbed onto *Bifidobacterium longum*, achieving a high binding efficiency of 98.67%. The study demonstrated that *Bifidobacterium longum* effectively facilitated the anchoring and accumulation of CL-ICG-PFH-NPs at the tumor site in a TNBC model. This targeted delivery significantly enhanced the therapeutic efficacy of HIFU and enabled multimodal imaging for early tumor detection and treatment monitoring. Notably, the group treated with B. longum + CL-ICG-PFH-NPs + HIFU exhibited a markedly higher volume of coagulative necrosis (180.158 ± 28.316 mm^3^) compared to the CL-ICG-PFH-NPs + HIFU group (58.639 ± 5.884 mm^3^), underscoring the synergistic benefits of this biohybrid system [47]. Table 3 represents the summary of *Bifidobacterium*-Mediated delivery of HIFU synergistic nanoparticles.

## 8. Limitations and Future Perspectives

*Bifidobacterium* itself can exhibit anti-cancer action by regulating the tumor microenvironment. Various mechanisms have been identified such as immune modulatory [48], regulating signaling molecules involved in cancer cell apoptosis [49,50], and higher production of short-chain fatty acids [51,52]. Further studies suggested that the *Bifidobacterium* enhances the action of immunotherapy and checkpoint inhibitor approaches for various cancers [51,52,53,54]. A study involving *Bifidobacterium infantis* combined with albumin-encapsulated doxorubicin nanoparticles (Bif@BDC-NPs) demonstrated that *Bifidobacterium infantis* alone induced 2.3 times more cell death in 4T1 breast cancer cells compared to the control group. Additionally, in hepatocellular carcinoma cell lines, *Bifidobacterium infantis* triggered apoptosis levels that were 2.04-fold higher in Huh7 cells, and 1.23-fold higher in HepG2 cells relative to controls [15]. In the endostatin gene therapy study, monotherapy of *Bifidobacterium adolescentis* resulted in a 23.1% inhibition of liver tumor growth. Further, the treatment with genetically engineered *Bifidobacterium adolescentis* carrying the endostatin gene achieved a significantly higher tumor growth inhibition of 69.9% [26]. In the in vivo study of CS-L-PGA-SN38 NPs/*B*, monotherapy of *Bifidobacterium bifi* also showed significant tumor volume inhibition in the colorectal tumor model [40]. It is evident that *Bifidobacterium* functions not only as a carrier for targeted delivery of anticancer drugs to tumor sites but also exhibits inherent anticancer activity by inducing apoptosis in cancer cells. Ongoing clinical trials are currently investigating the role of *Bifidobacterium* in modulating responses to various cancer treatments (NCT06772090, NCT06428422).

Furthermore, regarding the safety profile of *Bifidobacterium*, studies have shown that it does not induce hemolysis or apoptosis in normal hepatocytes or pulmonary cells [15]. In animal models, treatment with *Bifidobacterium* caused no organ damage, as evidenced by the absence of histopathological changes in the heart, liver, spleen, and lungs, along with normal kidney function in mice [15]. Wu et al. conducted an acute toxicity study on *Bifidobacterium bifidum* and observed no significant changes in body weight or morphological abnormalities in the organs of the treated group [40]. Further, in a study investigating the delivery of HIFU synergists using *Bifidobacterium bifidum*, no significant differences were observed in routine blood and biochemical test results in a TNBC mouse model after 14 days of synergist administration [46]. These studies clearly demonstrate that *Bifidobacterium* does not induce toxicity in normal cells or organs.

**Figure 3:**
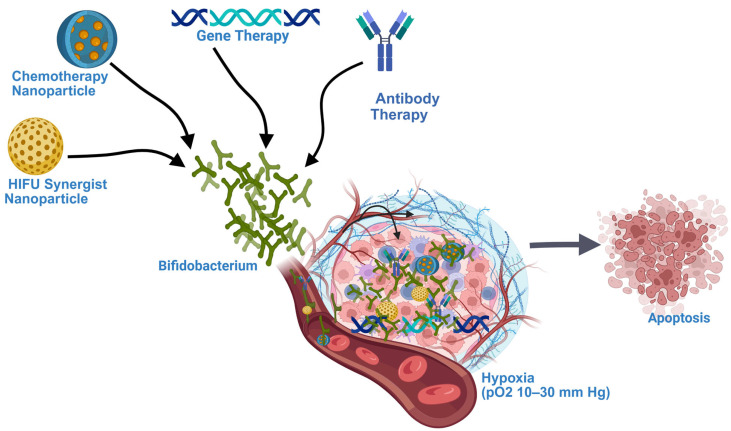
Illustration of *Bifidobacterium* colonization within the tumor microenvironment and its role in delivering Gene therapy, Chemotherapy nanoparticles, Immunotherapy, and high-intensity focused ultrasound (HIFU) synergistic nanoparticles.

It is well established that *Bifidobacterium* species preferentially colonize in the hypoxic environments of solid tumors. Additionally, their acid-resistant nature enables them to thrive in the acidic microenvironment of cancer cells. A growing body of pre-clinical research has demonstrated the promising potential of various *Bifidobacterium* strains as targeted delivery vehicles for cancer therapies. Notably, several studies have successfully employed *Bifidobacterium*-mediated systems to deliver nano-formulated chemotherapeutic agents such as doxorubicin and paclitaxel directly to tumor cells, enhancing therapeutic efficacy while minimizing systemic toxicity (Table 2) [15,38,39,40,41]. In addition to nano-formulated chemotherapeutics delivery, *Bifidobacterium* has been utilized for the targeted transport of gene and immunotherapy, particularly those aimed at inhibiting angiogenesis and promoting apoptosis in tumor tissues [25,26,27,28,29,30,31,32,33,34] (Table 1) (Figure 1 and Figure 3). One of the most significant advancements in this field is the use of *Bifidobacterium* to deliver nanoparticles that act as enhancers for HIFU therapy (Table 3). This approach has shown considerable promise in enhancing HIFU-mediated tumor ablation, especially in the treatment of triple-negative breast cancer (TNBC) [42,43,44,45].

One significant limitation it can occur is the potential inhibition of *Bifidobacterium* by anti-cancer drugs. However, a study found that even at concentrations higher than those typically found in plasma, several chemotherapy agents, including capecitabine, cyclophosphamide, docetaxel, erlotinib, gefitinib, irinotecan, and paclitaxel, did not inhibit the growth of 34 species of *lactic acid bacteria* (LAB), *Bifidobacterium*, or other intestinal microbes. For all *Bifidobacterium* species tested, the minimum inhibitory concentrations (MICs) were greater than 128 µg/mL [55].

Further research is essential to deepen our understanding and critically evaluate the advantages and limitations of *Bifidobacterium*-mediated cancer therapies. Currently, clinical trials investigating these approaches remain limited. A clinical study named “A Phase I/II Safety, Pharmacokinetic, and Pharmacodynamic Study of APS001F with Flucytosine (5FC) and Maltose for the Treatment of Advanced and/or Metastatic Solid Tumors” was initiated in 2012 including patients with advanced and/or metastatic solid tumors. However, no results were published from this study. The FDA has recently approved two microbiota-based therapies Rebyota™ and Vowst™ for the treatment of recurrent *Clostridioides difficile* infection. As *Bifidobacterium*-related clinical trials progress, the manufacturing processes and analytical methodologies for these treatments may require validation in accordance with existing regulatory frameworks.

Clinical studies should be initiated to explore the colonization patterns of different *Bifidobacterium* species across various solid tumor types. Moreover, it is crucial to identify the factors and external stimuli, such as specific energy sources, that can enhance *Bifidobacterium* proliferation within the tumor microenvironment. Research should focus on optimizing conditions that promote bacterial colonization, including the use of targeted nutritional and metabolic support. Pre-clinical studies have shown that lactulose, a disaccharide composed of galactose and fructose, can significantly promote *Bifidobacterium* growth in animal models. Importantly, lactulose is not metabolized by mammalian cells, making it a selective energy source for bacterial growth [56]. However, for clinical application, it is necessary to identify and validate suitable energy or nutritional substrates that are both safe and effective in human subjects.

Several studies have reported limited bacterial colonization at tumor sites, which may be attributed to restricted vascular access [57]. Blocked or poorly perfused blood vessels within tumors can hinder bacterial entry [55]. To address this, vasodilator therapy has been proposed as a strategy to enhance *Bifidobacterium* colonization. For instance, studies demonstrated that tumor colonization by *Lactobacillus casei* was significantly improved through the administration of vasodilators such as nitric oxide and the angiotensin-converting enzyme (ACE) inhibitor enalapril [58,59]. These findings suggest that vasodilators may enhance the Enhanced Permeability and Retention (EPR) effect, thereby facilitating greater bacterial accumulation at tumor sites, a strategy that could be applicable to *Bifidobacterium* as well. Another factor contributing to reduced bacterial colonization is the clearance of bacteria by the reticuloendothelial system (RES), which can eliminate circulating bacteria before they reach the tumor microenvironment [60].

## 9. Conclusions

The effectiveness of cancer treatments can be limited by non-targeted delivery methods. Recently, there has been growing interest in developing tumor-targeted drug delivery strategies for solid tumors. Among these, *Bifidobacterium*-mediated delivery shows significant promise, as it not only enhances treatment efficacy but also minimizes side effects. This approach has been explored in various therapeutic modalities, including immunotherapy, gene therapy, chemotherapy, and high-intensity focused ultrasound (HIFU) enhancers (Figure 3). However, further research is required to address current limitations and to optimize bacterial colonization for improved therapeutic outcomes.

## Figures and Tables

**Table 1:** *Bifidobacterium*-mediated gene therapy delivery.

Gene	Mode of Action	*Bifidobacterium*	Cancer	Observation (In Vivo)	Reference
*Cytosine deaminase gene (CDG)*	Conversion of the 5-fluorocytosine (5-FC) into active 5-FU	*B. longum*	Lung, Breast		[24]
*Cytosine deaminase gene (CDG)*	Conversion of the 5-fluorocytosine (5-FC) into active 5-FU	*B. breve (Higher CDG activity compared to B. longum)*	Lung		[25]
Endostatin	Angiogenesis inhibitor— downregulation of bFGF and VEGF	*B. adolescentis*	Liver	Inhibition of Tumor growth (69.9%)	[26]
Tumstatin	Angiogenesis inhibitor and promote apoptosis	*B. longum*	Colorectal	Inhibition of Tumor growth (75.21%)	[28]
Enterotoxin (C-terminal fragment of the Clostridium perfringens (C-CPE) (Claudin-4 blocker)	Claudin-4 inhibition—inhibits proliferation	*B. longum*	Breast (TNBC)	Inhibition of Tumor growth (49.4%)	[29]
Interleukin-24	Promote apoptosis and/or autophagy	*B. breve*	Head and neck squamous		[30]
*Kinase insert domain receptor (sKDR)*	Angiogenesis inhibitor (VEGF-mediated)	*B. infantis*	Lung	Inhibition of Tumor growth and higher survival (*p* ≤ 0.05)	[32,33]

**Table 2:** *Bifidobacterium*-Mediated Nanoparticle Formulated Chemotherapy Delivery.

Chemotherapy	Biohybrid	*Bifidobacterium*	Cancer	Observation (In Vivo)	Reference
Doxorubicin	Albumin-encapsulated doxorubicin coated with chitosan (Bif@BDC-NPs)	*B. infantis*	Breast	Inhibited tumor growth (94%)	[38]
Doxorubicin	Doxorubicin-loaded bovine serum albumin (Bif@DOX-NPs)	*B. infantis*	Breast	Inhibited tumor growth and prolonged the median survival of the tumor-bearing mice to 69 days	[15]
Paclitaxel	Polydopamine (PDA)-coated paclitaxel (Bif@PDA-PTX-NPs)	*B. infantis*	Lung	Inhibited tumor growth and prolonged the survival of tumor-bearing mice.	[39]
Irinotecan (CPT-11)-SN38	Poly-L-glutamic acid SN38 (CS-L-PGA-SN38 NPs/B. bifi)	*B. bifidum*	Colorectal	Inhibited tumor growth (80%)	[40]
Doxorubicin & Endostatin	Iron alginate (FeAlg) gel with doxorubicin and endostatin (BI-ES-FeAlg/DOX)	*B. infantis*	Colorectal	Inhibited tumor growth (82%)	[41]

**Table 3:** *Bifidobacterium*-Mediated HIFU Synergistic Nanoparticles Delivery.

HIFU Synergist Nanoparticle	Biohybrid	*Bifidobacterium*	Observation (In Vivo)	Reference
Perfluorohexane (PFH) and superparamagnetic iron oxides (SPIO, Fe_3_O_4_) with cationic lipid (CL)	*B. bifidum* + PFH@CL/Fe_3_O_4_ NPs	B. bifidum	Higher coagulative necrosis and apoptosis	[43]
Aptamers CCFM641-5-functionalized Perfluorohexane (PFH) loaded poly(lactic-co-glycolic acid	*Bifidobacterium*+ AP-PFH/PLGA	*B. bifidum*	Tumor growth inhibition and prolong the survival (60 days) compared to docetaxel (30 days)	[44]
PEGylated cationic lipid nanoparticles (CL-NPs)	*B. longum* + CL-NPs	*B. longum*	Higher coagulative necrosis and apoptosis	[45]
Polyethylenimine (PEI)-modified poly(lactic-co-glycolic acid) nanoparticles loaded with sodium bicarbonate	*B. bifidum* + PEI-PLGA-NaHCO_3_ NPs	*B. bifidum*	Higher coagulative necrosis and apoptosis	[43]
Cationic lipid nanoparticles co-loaded with indocyanine green (ICG) and perfluorohexane (PFH)	*B. longum* + CL-ICG-PFH-NPs	*B. longum*	Higher coagulative necrosis volume and apoptosis	[46]
